# Electronic cigarette use and harm reversal: emerging evidence in the lung

**DOI:** 10.1186/s12916-015-0298-3

**Published:** 2015-03-18

**Authors:** Riccardo Polosa

**Affiliations:** Centro per la Prevenzione e Cura del Tabagismo (CPCT), Azienda Ospedaliero-Universitaria “Policlinico-Vittorio Emanuele”, Università di Catania, Catania, Italy; Dipartimento di Medicina Clinica e Sperimentale – Università di Catania, Azienda Ospedaliero-Universitaria “Policlinico-Vittorio Emanuele”, Università di Catania, Catania, Italy; UOC di Medicina Interna e d’Urgenza, Edificio 4, Piano 3, AOU “Policlinico-V. Emanuele”, Via S. Sofia 78, 95123 Catania, Italy

**Keywords:** E-cigarette, E-vapor products, Harm reversal, Lung function, Respiratory system, Smoking cessation, Tobacco harm reduction

## Abstract

Electronic cigarettes (ECs) have been rapidly gaining ground on conventional cigarettes due to their efficiency in ceasing or reducing tobacco consumption, competitive prices, and the perception of them being a much less harmful smoking alternative. Direct confirmation that long-term EC use leads to reductions in smoking-related diseases is not available and it will take a few decades before the tobacco harm reduction potential of this products is firmly established. Nonetheless, it is feasible to detect early changes in airway function and respiratory symptoms in smokers switching to e-vapor. Acute investigations do not appear to support negative respiratory health outcomes in EC users and initial findings from long-term studies are supportive of a beneficial effect of EC use in relation to respiratory outcomes. The emerging evidence that EC use can reverse harm from tobacco smoking should be taken into consideration by regulatory authorities seeking to adopt proportional measures for the e-vapor category.

## Background

The electronic cigarette (EC) has been rapidly gaining ground on conventional cigarettes and could surpass consumption of conventional cigarettes within the next decade, according to some prediction analyses [1]. The growing popularity of ECs proves that many adult smokers are keen on using an alternative technologic form of smoking to reduce cigarette consumption or quit smoking and to relieve tobacco withdrawal symptoms [2]. Data from internet surveys [2,3] and clinical trials [4,5] have shown that ECs may help smokers quit or reduce their tobacco consumption. Moreover, the popularity of ECs appears to be associated with the fact that they can be used in many smoke-free areas, their prices are competitive, and they are perceived as a much less harmful smoking alternative [3,6].

Vapor toxicology under normal conditions of use is by far less problematic than that of conventional cigarettes [7], and exclusive EC users have significantly lower urine levels of tobacco smoke toxicants and carcinogens compared to cigarette smokers [8]. Thus, smokers completely switching to regular EC use are likely to gain significant health benefits.

Although a reduction in smoking-related diseases from long-term EC use can be inferred by the positive findings on Swedish snus (a tobacco harm reduction product consisting of refined oral tobacco which is low in nitrosamines) [9], direct confirmation is not available and it will take a few decades before a reduction in individual and population health outcomes due to the regular use of e-vapor products can be firmly established. Nonetheless, it is feasible to detect early changes in airway function and respiratory symptoms in smokers switching to e-vapor.

In this commentary, I discuss the emerging potential of ECs for harm reversal with a specific focus on the respiratory system.

## Health outcomes and the respiratory system

The lung is the primary target of the harmful effects of several airborne pollutants and cigarette smoke. Likewise, considering that inhalation is the exposure mechanism for EC use, the respiratory system is also the logical target for investigating any potential harmful effects of chemicals in the e-vapor.

Prospective clinical studies of well-characterized EC users would be the most informative to investigate respiratory health outcomes; however, such studies are quite demanding due to several methodological, logistical, ethical, and financial challenges. In particular, to address the potential of future disease development, hundreds of users would need to be followed for a substantial number of years before any conclusions could be made. A much less challenging approach is to explore cytotoxicity levels, mutagenicity, genotoxicity, oxidative stress, and inflammatory responses in human lung epithelial cell lines. However, these *in vitro* approaches also have inherent flaws; findings cannot be directly applied in human *in vivo* studies due to the inability to test the normal consumption exposure conditions of e-vapor products, the fact that standards for vapor production and exposure protocols have not been clearly defined, and the risk of over- or underestimating the interpretation of the toxic effects in these investigational models. Consequently, it is not surprising to find a divergence in the literature, with some authors reporting little or no injury [10], whereas others describing much higher level of toxicity and inflammatory responses despite using same cell lines [11]. Overall, despite the inconsistent and contradictory results, most acute *in vitro* studies are simply suggestive of non-specific irritant effects from e-vapor exposure. This is consistent with findings from internet surveys and clinical trials reporting transient throat irritation, dry cough, and other symptoms of respiratory irritation in some smokers when switching to ECs (reviewed in [7]).

Symptoms of irritation may occur in EC users hypersensitive to propylene glycol present in the e-vapor, but the possibility of unknown contaminants or byproducts contained in the e-vapor causing similar irritant effects cannot be discounted [12]. Likewise, a prompt defensive response against irritants from e-vapor inhalation is the most likely cause for the immediate physiologic changes detected with highly sensitive respiratory functional tests as reported by Vardavas et al. [13]. The question of whether such an irritation could translate into clinically meaningful lung disease remains unanswered, and there certainly is no evidence to date to suggest that there are any clinically significant adverse lung effects, at least acutely.

Moreover, it must be noted that the reported 16% decrease in exhaled nitric oxide levels (i.e., 2.1 ppb in absolute terms) and 11% increase in peripheral flow resistance by impulse oscillometry (i.e., 0.025 kPa/L/s in absolute terms) from baseline after using an EC for 5 minutes were so small and well within test variability, that it is unlikely to have any clinical relevance [14,15]. Moreover, no significant changes were detected by less sensitive respiratory function parameters (including forced expiratory volume in the first second (FEV1), forced vital capacity (FVC), FEV% (FEV1/FVC index), and peak expiratory flow (PEF)) after EC use. Lack of a significant effect on exhaled nitric oxide and airflow obstruction as measured by FEV1, FVC, FEV%, and PEF after short-term EC use has also been confirmed in a more recent study [16]. Finally, switching to EC use universally leads to a near-normalization in toxic levels of exhaled carbon monoxide (reviewed in [7]).

The very few studies mentioned above, which have focused on the acute effect of ECs on lung function, do not appear to support negative respiratory health outcomes in EC users. Nonetheless, only large and carefully conducted studies evaluating the long-term effects of these products will provide a definite answer regarding their impact on lung health.

As mentioned earlier, it would take hundreds of well-characterized EC users to be followed prospectively for a substantial number of years and very large funding to properly address the harm potential of ECs. At the University of Catania, we have structured an integrated clinical research program characterized by a minimalist approach entailing either highly sensitive respiratory functional tests to detect early changes of subclinical injury in ‘healthy’ smokers switching to EC or less sensitive but more robust respiratory function investigations to explore changes in EC users with preexisting lung disease. The initial findings are promising and generally supportive of a beneficial effect of EC use in relation to respiratory outcomes, both in health and disease.

Long-term changes in lung function have been monitored for up to 1 year in a large group of ‘healthy’ smokers who were invited to quit or reduce their tobacco consumption by switching to a first generation EC. Significant early positive changes from baseline of a sensitive measure of obstruction in the more peripheral airways (i.e., forced expiratory flow measured between 25% and 75% of FVC) were already detected at 3 months after switching in those who completely gave up tobacco smoking, with steady progressive improvements being observed also at 6 and 12 months (Polosa R, unpublished observation).

Asthma and chronic obstructive pulmonary disease (COPD) are progressive diseases characterized by persistent inflammatory and remodeling responses of the airways causing respiratory symptoms and progressive decline in lung function [17,18]. Although it is well-established that the inflammatory response to cigarette smoke plays a key role in COPD pathogenesis, increased morbidity and mortality have been reported in asthmatic individuals who smoke and quitting can significantly improve asthma symptoms and lung function [19]. Consequently, smokers with preexisting asthma and COPD may benefit from regular EC use. In the only clinical study conducted to ascertain efficacy and safety of EC use in asthma, substantial improvements in respiratory physiology and subjective asthma outcomes have been reported [20]. Exposure to e-vapor in this vulnerable population did not trigger any asthma attacks.

To date, no formal efficacy and safety assessment of EC use in COPD patients has been conducted. There is only evidence from a case series of three inveterate smokers with COPD, who eventually quit tobacco smoking on their own by switching to an EC [21]. Significant improvement in quality of life and reduction in the number of disease exacerbations were noted. EC use was well tolerated with no reported adverse events.

The reported improvements of respiratory patients who have become regular ECs users are consistent with findings from a large internet survey of regular EC users diagnosed with asthma and COPD [2]. An improvement in symptoms of asthma and COPD after switching was reported in 65.4% and 75.7% of the respondents, respectively. Compared to dual users, improvement in symptoms of asthma and COPD were more often reported by exclusive EC users. After switching, medications were stopped in 460/2,498 (18.4%) respondents with asthma and COPD. Worsening after switching was only reported in 1.1% of the asthmatics and in 0.8% of the COPD respondents. Taken together, these findings provide emerging evidence that EC use can reverse harm from tobacco smoking.

## Conclusions and implications for policymaking

Compared to combustible cigarettes, e-vapor products are at least 96% less harmful and may substantially reduce individual risk and population harm [22]. Future research will better define and further reduce residual risks from EC use to as low as possible by establishing appropriate quality control and standards. Although large longitudinal studies are warranted to elucidate whether ECs are a less harmful alternative to tobacco cigarettes and whether significant health benefits can be expected in smokers who switch from tobacco to ECs, the emerging evidence that EC use can reverse harm from tobacco smoking should be taken into consideration by regulatory authorities seeking to adopt proportional measures for the e-vapor category [23].

## Abbreviations

COPD: Chronic obstructive pulmonary disease
ECs: Electronic cigarettes
FEV%: FEV1/FVC index
FEV1: Forced expiratory volume in the first second
FVC: Forced Vital Capacity
PEF: Peak Expiratory Flow
